# Comparison of narrowband ultraviolet B with psoralen plus ultraviolet A phototherapy for patients with early-stage mycosis fungoides: a systematic review and meta-analysis

**DOI:** 10.1093/skinhd/vzag055

**Published:** 2026-06-15

**Authors:** Serish Bano, F N U Sidra, F N U Venjhraj, Mutahira Asif, Aghna Iman, F N U Fatima, Meva Ram, Aymar Akilimaili

**Affiliations:** Department of Medicine, Shaheed Mohtarma Benazir Bhutto Medical College Lyari, Karachi, Pakistan; Department of Medicine, Shaheed Mohtarma Benazir Bhutto Medical College Lyari, Karachi, Pakistan; Department of Medicine, Shaheed Mohtarma Benazir Bhutto Medical College Lyari, Karachi, Pakistan; Department of Medicine, Shaheed Mohtarma Benazir Bhutto Medical College Lyari, Karachi, Pakistan; Department of Medicine, Shaheed Mohtarma Benazir Bhutto Medical College Lyari, Karachi, Pakistan; Federal Medical College, SZABMU, Islamabad, Pakistan; Department of Medicine, Shaheed Mohtarma Benazir Bhutto Medical College Lyari, Karachi, Pakistan; Department of Research, Medical Research Circle (MedReC), Goma, Democratic Republic of the Congo

## Abstract

**Background:**

The most prevalent cutaneous T-cell lymphoma is mycosis fungoides (MF), managed in early stages with psoralen plus ultraviolet A (PUVA) or narrowband ultraviolet B (NB-UVB) phototherapy.

**Objectives:**

To evaluate the therapeutic effectiveness and side effect profiles of PUVA vs. NB-UVB in patients with early-stage MF.

**Method:**

A systematic review was conducted, incorporating data from nine studies sourced from PubMed, Cochrane and Google Scholar. Eligible studies were those that simultaneously evaluated PUVA and NB-UVB, enrolling at least 10 adult participants per group with histologically confirmed early-stage (IA–IIA) MF and reported treatment outcomes. Exclusions were advanced disease, paediatric patients, noncomparative or nonrelevant treatments, small sample sizes or lack of outcome data. No language restrictions were applied. Key outcomes included any response, complete response, partial response, treatment failure, relapse-free interval and adverse effects.

**Results:**

A total of 923 patients (age range 33–71 years; 52.5% men) were included: 556 treated with PUVA and 367 with NB-UVB. Any response was seen in 90.5% of those treated with PUVA vs. 88.3% of patients who received NB-UVB [odds ratio (OR) 1.18, 95% confidence interval (CI) 0.73–1.90; *P* = 0.50]. Complete response rates were 72% for those treated with PUVA and 61.8% for those treated with NB-UVB (OR 1.12, 95% CI 0.60–2.07; *P* = 0.73). Partial response was observed in 19.2% of patients who received PUVA and 28.0% of those who received NB-UVB (OR 0.87, 95% CI 0.45–1.69; *P* = 0.69). Treatment failure rates were lower with PUVA (9.7%) than with NB-UVB (12.6%) (OR 0.81, 95% CI 0.49–1.33; *P* = 0.41). Patients who received PUVA had a significantly longer median relapse-free interval (hazard ratio 1.94, 95% CI 1.07–3.51; *P* < 0.01). Adverse effects, including erythema, nausea, pruritus, burning, hyperpigmentation, pain, phototoxic reactions and polymorphic light eruption, showed no significant differences between groups.

**Conclusions:**

Both PUVA and NB-UVB are effective, but PUVA may be preferred when sustained remission is the primary therapeutic goal.

## Teaser

This systematic review compares PUVA and NB-UVB phototherapy for early-stage mycosis fungoides. Both treatments showed comparable response rates, but PUVA was associated with a significantly longer relapse-free interval, suggesting potential advantages in long-term disease control.

What is already known about this topic?

- Psoralen plus ultraviolet A (PUVA) and narrowband ultraviolet B (NB-UVB) phototherapy to treat early-stage mycosis fungoides are both effective, with PUVA demonstrating a higher complete response rate.
- However, no significant differences have been observed in overall response rates or adverse events, and the quality of studies is generally low to moderate.
- Previous findings suggested PUVA as a potential alternative to NB-UVB, but there is uncertainty regarding long-term outcomes and relapse rates.

What does this study add?

- This updated meta-analysis includes a larger pooled patient population and incorporates newer studies, enhancing the statistical power and robustness of comparisons.
- While overall and complete response rates between PUVA and NB-UVB remain statistically comparable, this study identifies a significantly longer relapse-free interval with PUVA therapy.
- This finding provides important additional insight into long-term disease control, reinforcing the potential advantage of PUVA in sustaining remission for patients with early-stage mycosis fungoides.

Mycosis fungoides (MF) is the most prevalent variant of cutaneous T-cell lymphoma. It predominantly affects older adults and has a slight male predominance (male-to-female ratio of approximately 2:1).^1^ Early-stage MF skin lesions typically appear as patches or plaques, and primarily appear on parts of the skin that are not exposed to sunlight, such as the chest and buttocks. It is uncommon for these lesions to evolve into tumours involving the lymphatic ­system and internal organs. Most cases of MF are diagnosed early (IA–IIA), which is typically linked to a good prognosis.^2^ Treatment strategies for MF are guided by the disease stage. Skin-directed treatments such topical corticosteroids, topical chemotherapy, oral and topical retinoids, methotrexate and several types of phototherapy are usually the mainstay of treatment for early-stage MF. In contrast, individuals with stage IIB–IVB Sézary syndrome or advanced-stage MF often exhibit limited responsiveness to conventional treatments. These patients often require more aggressive systemic approaches, such as extracorporeal photopheresis, radiation, chemotherapy or allogeneic stem cell transplantation.^3^

Phototherapy is a key component of the management of patients with early-stage MF. The most frequently employed techniques are psoralen plus ultraviolet A (PUVA) phototherapy, which combines topical or systemic psoralen with ultraviolet (UV) irradiation between 320 and 340 nm, and narrowband UVB (NB-UVB) therapy, which uses UV radiation at a wavelength of 311 nm. The fundamental mechanisms of action and electromagnetic and biological characteristics of PUVA and NB-UVB are distinct. UVA’s greater wavelength allows it to penetrate deeper into the dermis, which is believed to improve the initial reaction and extend the duration of disease-free living. Therefore, PUVA might be more beneficial for people who have thick plaques or who are resistant to NB-UVB therapy. Additionally, psoralen directly interacts with DNA to cause damage, producing reactive oxygen species that harm cell organelles.^4,5^ Additionally, it has been proposed that UVA causes apoptotic cascades through p53-independent mechanisms of programmed cell death.^4,5^ It has been demonstrated that PUVA’s phototoxic actions specifically target malignant T cells.^4,6,7^ Although the exact processes of NB-UVB therapy in MF are unknown, it is believed that they entail interfering with immunity through Langerhans cells. The cells’ capacity to deliver antigens is inhibited, and the cytokines tumour necrosis factor, interleukin (IL)-2 and IL-6 are elevated.^5,8^ T-cell apoptosis may also play a role in MF by inhibiting neoplastic activity.^9^

This work builds on the prior meta-analysis by Phan *et al*.,^10^ which included seven studies and mainly assessed response rates. By adding newer studies and evaluating outcomes such as relapse-free interval, treatment failure and adverse events, our review provides updated evidence on long-term disease control in early-stage MF. The objective was to compare the efficacy and safety of PUVA and NB-UVB in this setting.

## Materials and methods

This meta-analysis and systematic review adhered to the PRISMA framework and established Cochrane standards.^11^

### Literature search and search strategy

A thorough literature search was conducted by the study team using a number of databases, including the Cochrane Library, PubMed and Google Scholar. Studies published from the inception of each database up to March 2025 were identified using specific keywords (Narrowband Ultraviolet B OR Narrowband UV-B OR NB-UVB phototherapy OR psoralen-UV-A OR psoralen ultraviolet OR PUVA phototherapy) AND (early-stage mycosis fungoides OR early-stage MF). In addition, we reviewed the reference lists of relevant studies to identify further articles for inclusion in our study library. A comprehensive search string with all relevant keywords used during the literature search is provided in Table 1.

**Table 1:** Search strategy used in each database

Search string	Database	Number of papers retrieved
(((((((Narrowband Ultraviolet B) OR (Narrowband UV-B)) OR (NB UVB phototherapy)) OR (psoralen UV-A)) OR (psoralen ultraviolet)) OR (PUVA phototherapy)) AND (early-stage mycosis fungoides)) OR (early-stage MF)	PubMed	1386
(((((((Narrowband Ultraviolet B) OR (Narrowband UV-B)) OR (NB UVB phototherapy)) OR (psoralen UV-A)) OR (psoralen ultraviolet)) OR (PUVA phototherapy)) AND (early-stage mycosis fungoides)) OR (early-stage MF)	Cochrane Library	687
(((((((Narrowband Ultraviolet B) OR (Narrowband UV-B)) OR (NB UVB phototherapy)) OR (psoralen UV-A)) OR (psoralen ultraviolet)) OR (PUVA phototherapy)) AND (early-stage mycosis fungoides)) OR (early-stage MF)	Google Scholar	10

NB-UVB, narrowband ultraviolet B; PUVA, psoralen plus ultraviolet A.

### Selection criteria

Studies were considered eligible for inclusion in this systematic review and meta-analysis if they directly compared cohorts or cross-sectional groups of patients receiving PUVA phototherapy with those treated using NB-UVB phototherapy. The inclusion criteria required that studies be either cohort or cross-sectional in design, involve patients with histologically confirmed early-stage MF (IA to IIA), compare PUVA (oral or bath) with NB-UVB, include a minimum of 10 participants per treatment group and report therapeutic response outcomes. Retrospective cohort and cross-sectional studies were included and analysed together because of limited available data; separating them would have further reduced the statistical power. Studies were excluded if they involved patients with stage IIB or more advanced disease (including tumours, erythroderma or involvement of lymph nodes requiring systemic treatment), included paediatric populations, had fewer than 10 patients per treatment group, compared PUVA with treatments other than NB-UVB (e.g. interferon-α), were noncomparative in nature (e.g. case reports or conference abstracts) or did not report outcomes of interest. No language restrictions were applied.

### Data extraction and quality appraisal

The articles’ text, tables and illustrations were all used to extract the data. All retrieved articles were independently examined by two investigators (M.R. and S.B.). Discussion and agreement were used to settle disagreements between the two reviewers. For consistency, outcomes were defined as follows: any response = ­complete response (CR) + partial response (PR); CR = 100% clearance of skin lesions; PR = ≥50% but <100% clearance of lesions; treatment failure = <50% improvement or disease progression;^12^ disease recurrence = reappearance or progression of lesions after an initial response;^13^ and adverse events = ­treatment-related side effects as reported in the original studies (e.g. erythema, nausea, pruritus, phototoxicity, hyperpigmentation and pain).^14^ Outcomes were assessed at the end of treatment or at the last reported ­follow-up. Treatment failure was analysed separately in addition to overall, complete and partial responses, as it directly reflects nonresponders and is clinically relevant for identifying patients who may require escalation of therapy.

When the study provided medians and interquartile ranges rather than means and standard deviations (SDs), we used Hozo *et al*.’s methodology to estimate the means and SDs.^15^ Kaplan–Meier plots without a hazard effect or 95% confidence interval (CI) were determined using Tierney *et al*.’s methods.^16^ The quality of the studies was evaluated using the Cochrane Collaboration tool.

### Statistical analysis

Statistical analysis was carried out using RevMan (version 5.4.1; Cochrane Collaboration, London, UK). The Mantel–Haenzel technique was used to construct odd ratios (ORs) for dichotomous outcomes, and the hazard ratio (HR) with 95% CIs were applied for time-to-event outcomes (e.g. relapse-free survival). Cross-sectional studies (e.g. Zengarini *et al*.^17^) were excluded from HR analysis. A random-effects model was used for all analyses. Higgins’ *I*^2^ statistic was used to assess heterogeneity; modest heterogeneity is represented by *I*^2^ values between 25% and 50%, moderate heterogeneity by values between 50% and 75%, and substantial heterogeneity by values >75%. A sensitivity analysis was conducted for outcomes showing high heterogeneity to identify potential sources. Subgroup analyses were prespecified by disease stage (IA vs. IB), given the clinical rationale that treatment response may differ by stage severity. Throughout the analysis, a *P*-value of <0.05 was regarded as statistically significant.

## Results

After a thorough search of the literature, a total of 2083 studies were found. Following the elimination of duplicate records, 1390 studies remained. After additional exclusions based on retrieval, ineligibility, review and case report publications, nine studies remained for the review. Compared with the 2019 review by Phan *et al*.,^10^ this analysis additionally included the studies by Pattamadilok *et al*.^18^ and Zengarini *et al*.^17^ The PRISMA flowchart presents the summary of the literature search (Figure S1; see Supporting Information). Tables 2 and 3 provide a summary of the research and the baseline features of these studies. Most of the included studies were retrospective, while El-Mofty *et al*.^22^ presented a prospective design and Zengarini *et al*.^17^ a cross-sectional design. The quality evaluation of the included studies is summarized in Figure S2 (see Supporting Information). Most studies were rated as poor-to-­moderate in quality, mainly due to a lack of random sequence generation, allocation concealment and blinding. This overall moderate-to-low quality of evidence should be considered when interpreting the results.

**Table 2:** Characteristics of the included studies

Study	Study design	Inclusion criteria	PUVA frequency	NB-UVB frequency	No. of patients who received PUVA/no. of patients who received NB-UVB	Outcome
Ahmad 2007^19^	Retrospective	Topical therapy failed or patient unwilling to use it; stage IA–IIB MF (NB-UVB), stage IA–IVA MF (PUVA)	Twice weekly	Three times weekly	28/12	AR, CR, PR, FR
Almohideb 2017^20^	Retrospective cohort	Biopsy-proven stages IA and IB MF	Bath PUVA, twice weekly	Three times weekly	158/109	AR, CR, PR, FR, DR
Diederen 2003^21^	Retrospective	Histologically confirmed stages IA and IB MF	Twice weekly	Twice weekly	35/21	AR, CR, PR, FR
El-Mofty 2005^22^	Comparative	Stages IA or IB MF	Three times weekly	Three times weekly	10/10	AR, CR, PR
Nikolaou 2018^23^	Retrospective	Stages IA or IB MF	2–4 times weekly	2–4 times weekly	175/52	AR, CR, PR, FR, DR
Pattamadilo 2021^18^	Retrospective	Stages IA, IB, IIA, IIB, III	Twice weekly	Twice weekly	14/56	AR, CR, PR, FR
Ponte 2010^24^	Retrospective	Histologically confirmed stages IA, IB or IIA MF	Twice weekly	Three times weekly	95/19	AR, CR, PR, FR
Unal 2015^25^	Retrospective	Stages IA, IB or IIA MF	Three times weekly	Three times weekly	26/28	AR, CR, FR
Zengarini 2023^17^	Retrospective	Stages IA or IB MF	Three times weekly	Two or three times weekly	15/60	AR, CR, PR, FR, DR

AR, all response; CR, complete response; DR, disease recurrence; FR, failed response; MF, mycosis fungoides; NB-UVB, narrowband ultraviolet B; PR, partial response; PUVA, psoralen plus ultraviolet A.

**Table 3:** Baseline attributes of the patients in the included studies

Study	Treatment	*n*	Mean age (years)	Male (%)	Patch type (%)	Plaque type (%)	Stage IA MF (%)	Stage IB MF (%)	Stage IIA MF (%)	Follow-up (months)
Ahmad 2007^19^	PUVA	28	69.6	67.0	57.0	35.0	25.0	50.0	10.7	72.0
Ahmad 2007^19^	NB-UVB	12	58.7	58.0	58.3	33.3	50.0	33.3	8.3	84.0
Almohideb 2017^20^	PUVA	158	46.3	66.9	NA	NA	51.8	66.9	0.0	59.6
Almohideb 2017^20^	NB-UVB	109	44.2	33.1	NA	NA	48.2	33.1	0.0	22.3
Diederen 2003^21^	PUVA	35	53.0	57.0	NA	NA	NA	NA	NA	45.0
Diederen 2003^21^	NB-UVB	21	45.0	62.0	NA	NA	NA	NA	NA	77.0
El-Mofty 2005^22^	PUVA	10	33.0	60.0	80.0	20.0	10.0	90.0	0.0	NA
El-Mofty 2005^22^	NB-UVB	10	33.0	60.0	80.0	20.0	10.0	90.0	0.0	NA
Nikolaou 2018^23^	PUVA	175	57.8	60.6	40.6	59.4	57.7	42.3	0.0	50.1 (53.0)^a^
Nikolaou 2018^23^	NB-UVB	52	64.3	65.4	57.7	42.3	69.2	30.8	0.0	50.1 (53.0)^a^
Pattamadilok 2021^18^	PUVA	14	45.3	57.1	71.4	7.1	7.1	71.4	0.0	18.0
Pattamadilok 2021^18^	NB-UVB	56	41.4	42.9	37.5	5.4	26.8	53.6	5.4	9.1
Ponte 2010^24^	PUVA	95	59.0	48.4	NA	NA	32.6	60.0	7.4	NA
Ponte 2010^24^	NB-UVB	19	68.0	31.6	NA	NA	31.6	63.2	5.3	NA
Unal 2015^25^	PUVA	26	42.1	NA	NA	NA	30.8	46.2	23.1	50.7
Unal 2015^25^	NB-UVB	28	48.4	NA	NA	NA	35.7	53.6	10.7	22.5
Zengarini 2023^17^	PUVA	15	55.3	93.3	NA	NA	40.0	60.0	NA	12.0
Zengarini 2023^17^	NB-UVB	60	70.7	56.6	NA	NA	88.3	11.6	NA	60.0

MF, mycosis fungoides; NA, not available; NB-UVB, narrowband ultraviolet B; PUVA, psoralen with ultraviolet A. ^a^Mean (SD).

### Baseline characteristics

In this studies included in this meta-analysis, 367 patients received NB-UVB treatment and 556 patients received PUVA therapy. Mean patient age ranged from 33 to 70 years in the PUVA group and from 33 to 71 years in the NB-UVB group. In the PUVA group, 56.5% were men (*n* = 314/556), while in the NB-UVB group 46.6% were men (*n* = 171/367), a difference that was not statistically significant (*P* = 0.07). While plaque-type MF was more prevalent in the PUVA group (*n* = 117/227; 51.5%) than in the NB-UVB group (*n* = 31/130; 23.8%) (*P* = 0.05), patch-type MF was observed in 46.3% of patients who received PUVA (*n* = 105/227) and 50.8% of patients who received NB-UVB (*n* = 66/130) (*P* = 0.43). Regarding disease stage, stage IA MF was more prevalent in the NB-UVB group (*n* = 193/346; 55.8%) than in the PUVA group (*n* = 226/521; 43.4%), a statistically significant difference (*P* = 0.003). Conversely, stage IB MF was more frequent in the PUVA group (*n* = 272/521; 52.2%) than in the NB-UVB group (*n* = 136/346; 39.3%) (*P* = 0.02). Stage IIB disease was present in 9.8% (*n* = 16/163) of patients receiving PUVA and 7.0% (*n* = 8/115) of patients receiving NB-UVB (*P* = 0.35).

### Efficacy of treatment

**Figure 1:**
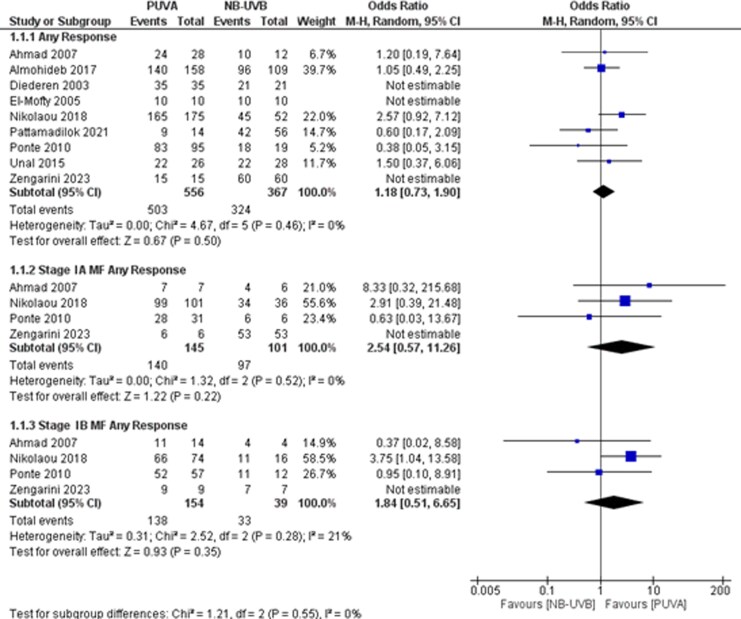
Forest plot of any response (complete or partial) in patients with early-stage mycosis fungoides treated with psoralen plus ultraviolet A (PUVA) or narrowband ultraviolet B (NB-UVB). CI, confidence interval.

‘Any response’ refers to the total of partial and full responses. Of the 556 patients who received PUVA, 503 (90.5%) with early-stage MF who received PUVA showed any response, while 324 of 367 (88.3%) patients who received NB-UVB did not achieve a response. The difference was statistically insignificant (OR 1.18, 95% CI 0.73–1.90; *P* = 0.50). Partial response occurred in 19.2% (PUVA) and 28.0% (NB-UVB) (*I*^2^ = 0%) (Figure 1). In the forest plots, the term ‘not estimable’ indicates outcomes where the effect size could not be calculated due to zero events or incomplete data reporting. A subgroup analysis was conducted for patients with stage IA MF, which showed a response in 140 of 145 (96.6%) patients who received PUVA and 97 of 101 (96.0%) patients who received NB-UVB. There was no statistically significant change (OR 2.54, 95% CI 0.57–11.26; *P* = 0.22, *I*^2^ = 0%). Some 138 of 154 patients (89.6%) treated with PUVA and 33 of 39 (85%) treated with NB-UVB showed any response for stage IB MF. Statistically, the difference was not significant (OR 1.84; 95% CI 0.51–6.65; *P* = 0.35, *I*^2^ = 21%).

**Figure 2:**
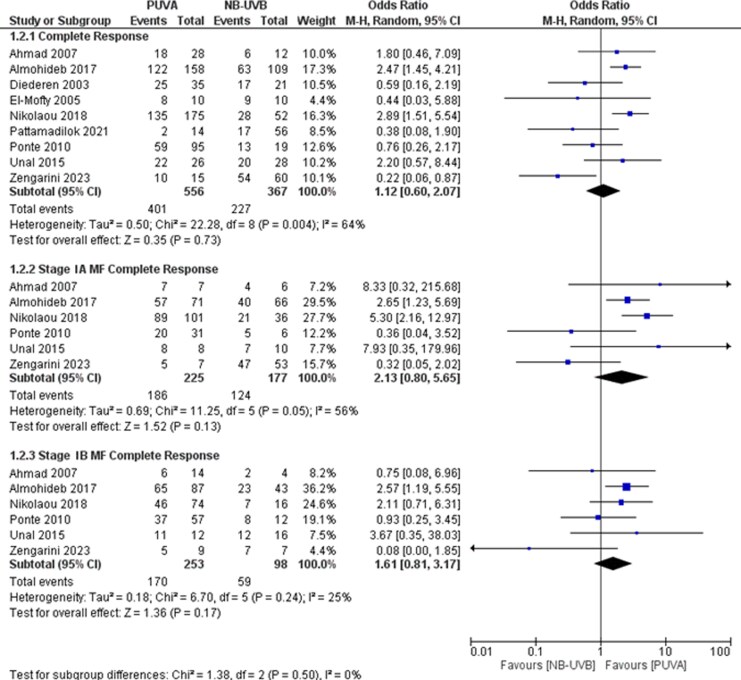
Forest plot of complete response in patients with early-stage mycosis fungoides (MF) treated with psoralen plus ultraviolet A (PUVA) or narrowband ultraviolet B (NB-UVB). CI, confidence interval.

The difference between the CR rates of 227 of 367 patients (61.8%) who received NB-UVB and 401 of 556 patients (72.1%) who received PUVA was not statistically significant (OR 1.12, 95% CI 0.60–2.07; *P* = 0.73, *I*^2^ = 64%) (Figure 2). In the subgroup analysis for stage IA MF, CRs were observed in 186 of 225 patients (82.7%) in the PUVA group and 124 of 177 patients (70.1%) in the NB-UVB group, which was also not statistically significant (OR 2.13, 95% CI 0.80–5.65; *P* = 0.13, *I*^2^ = 56%). Among patients with stage IB MF, 170 of 253 (67.2%) treated with PUVA achieved a CR compared with 59 of 98 (60%) treated with NB-UVB. This difference was not statistically significant (OR 1.61, 95% CI 0.81–3.17; *P* = 0.17, *I*^2^ = 25%).

In the PUVA group, 102 of 530 patients (19.2%) had PR outcomes vs. 95 of 339 patients (28.0%) in the NB-UVB group. (OR 0.87, 95% CI 0.45–1.69, *P* = 0.69, *I*^2^ = 64%) This difference was not statistically significant (Figure S3; see Supporting Information). In the subgroup of patients with stage IA MF, 20 of 101 patients (19.8%) treated with NB-UVB had PRs, compared with 19 of 146 patients (13.0%) treated with PUVA. This comparison also did not yield statistically significant results (OR 0.57, 95% CI 0.12–2.74; *P* = 0.48, *I*^2^ = 57%). Among stage patients with stage IB MF, 44 of 154 (28.6%) who received PUVA had a PR compared with 9 of 39 (23%) in the NB-UVB group. Again, the result was not statistically significant (OR 1.18, 95% CI 0.51–2.73; *P* = 0.69, *I*^2^ = 0%), indicating no observed heterogeneity.

Regarding failed treatment response (Figure S4; see Supporting Information), 53 of 546 patients (9.7%) who received PUVA experienced treatment failure compared with 45 of 357 patients (12.6%) who received NB-UVB. The difference was not statistically significant (OR 0.81, 95% CI 0.49–1.33; *P* = 0.41, *I*^2^ = 6%), suggesting no overall advantage of either treatment in preventing failure. However, subgroup analysis by disease stage revealed significant differences. In patients with stage IA MF, failed response was observed in 21 of 218 patients (9.6%) treated with PUVA compared with 33 of 124 patients (26.6%) treated with NB-UVB. This difference was statistically significant in favour of PUVA (OR 0.41, 95% CI 0.21–0.78; *P* = 0.007, *I*^2^ = 0%). Similarly, in those with stage IB MF, treatment failure was reported in 39 of 244 patients (16.0%) who received PUVA compared with 29 of 90 patients (32%) who received NB-UVB. This also showed a significant benefit for PUVA (OR 0.41, 95% CI 0.23–0.75; *P* = 0.004, *I*^2^ = 0%).

**Figure 3:**
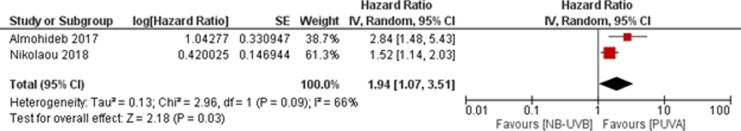
Forest plot of pooled hazard ratios of disease recurrence for early-stage mycosis fungoides treated with psoralen plus ultraviolet A (PUVA) or narrowband ultraviolet B (NB-UVB). CI, confidence interval.

PUVA therapy was associated with a relapse-free interval ranging from 10.0 to 45.2 months (median 33.4), notably longer than that observed with NB-UVB, which ranged from 5.2 to 24.5 months (median 14.9). Three studies^17,20,23^ reported statistically significant prolongation of relapse-free intervals with PUVA, while the others observed no notable difference between PUVA and NB-UVB. For disease recurrence, a total of two studies were included in the meta-analysis. The pooled HR for disease recurrence was 1.94 (95% CI 1.07–3.51; *P* = 0.003), suggesting a significant difference between PUVA and NB-UVB (Figure 3). In this context, an HR >1 indicates a higher probability of remaining relapse-free in the PUVA group compared with the NB-UVB group.

### Adverse effects

The incidence of adverse effects did not significantly differ between the PUVA and NB-UVB treatment groups. Rates of erythema were similar [32 of 308 (10.4%) vs. 9 of 93 (10%); *P* = 0.98], as were nausea [2 of 28 (7%) vs. 0 of 12 (0%); *P* = 0.59], pruritus [5 of 158 (3.2%) vs. 1 of 52 (2%); *P* = 0.93], burning sensation [20 of 130 (15.4%) vs. 3 of 40 (8%); *P* = 0.42], postinflammatory hyperpigmentation [1 of 210 (0.5%) vs. 2 of 73 (3%); *P* = 0.29] and pain [4 of 210 (1.9%) vs. 2 of 73 (3%); *P* = 0.73]. Similarly, no significant difference was found for phototoxic reactions [2 of 10 (20%) vs. 1 of 10 (10%); *P* = 0.54] or polymorphic light eruption [6 of 270 (2.2%) vs. 0 of 71 (0%); *P* = 0.68].

### Publication bias

To evaluate the risk of publication bias in studies comparing PUVA and NB-UVB for early-stage MF, a funnel plot was utilized. The distribution of data points appeared balanced, indicating that the likelihood of bias affecting the overall findings was insignificant (Figure S5; see Supporting Information).

## Discussion

In an updated meta-analysis, we compared the effects of PUVA and NB-UVB phototherapy on MF in its early stages. The two modalities did not differ statistically significantly in terms of adverse effects, CR, PR or any response. In contrast to NB-UVB (14.9 months), PUVA provided a longer median relapse-free interval (33.4 months) and was linked to noticeably decreased treatment failure rates in subgroups of patients with stage IA and IB MF.

Our updated meta-analysis revealed that PUVA and NB-UVB were equally effective in treating early-stage MF, with no discernible difference in total response rates (the sum of CRs and PRs). This aligns with findings by Phan *et al*.,^10^ who analysed data from 7 studies involving 778 patients and reported response rates of 90.9% for PUVA and 87.6% for NB-UVB. Although the difference was not statistically significant, our meta-analysis revealed that PUVA had numerically greater CR rates than NB-UVB. This supports the findings of Zengarini *et al*.,^17^ who reported CR rates of 90% for NB-UVB and 66.7% for PUVA in early-stage MF, with no significant difference. Similarly, Ahmad *et al*.^19^ studied 40 patients treated between 1980 and 2003 – 28 with PUVA and 12 with NB-UVB – and found CR rates of 64% for PUVA and 50% for NB-UVB. Among patients with stage IA and IB disease, CR rates were 62% for PUVA and 60% for NB-UVB, again indicating comparable efficacy.

PR, defined as a ≥50% reduction in lesion burden without complete clearance, remains clinically relevant in early MF. Our pooled analysis found no significant difference in PR rates between the two modalities. Ponte *et al*.^24^ reported nearly identical rates: 25.3% for PUVA and 26.3% for NB-UVB. Pattamadilok *et al*.^18^ also found comparable PR rates (50% vs. 44.6%), with no statistical difference. Our meta-analysis demonstrated that PUVA is associated with a lower treatment failure rate compared with NB-UVB in patients with early-stage MF. This finding is supported by previous meta-analyses by Phan *et al*.^10^

Growing evidence suggests that PUVA may offer superior long-term disease control over NB-UVB, being associated with longer relapse-free survival and lower recurrence rates. This advantage probably stems from the deeper skin penetration of UVA, allowing more effective targeting of dermal atypical T cells. For instance, Goyal *et al*.^26^ found that PUVA provided a longer disease-free interval than NB-UVB. The British Association of Dermatologists guidelines by Whittaker *et al*.^27^ also highlight PUVA’s role in sustaining remission in patients with MF. However, long-term safety remains an important consideration. Cumulative PUVA exposure has been associated with phototoxic reactions and photoageing, and long-term prospective studies have shown a significantly increased risk of cutaneous squamous cell carcinoma and, at very high cumulative doses, melanoma.^28,29^ These risks appear to be dose-dependent and emphasize the need for careful patient selection and ongoing dermatological monitoring, especially in younger patients who may require prolonged or repeated courses of therapy.

Our meta-analysis found no significant difference in adverse effects between PUVA and NB-UVB, including erythema, nausea, pruritus, phototoxic reactions, dyspepsia or pain, consistent with the findings of Phan *et al*.^10^ Many adverse effects, short- and long-term, may result from PUVA therapy. Burning, blistering, itching and redness are examples of short-term skin reactions. A higher incidence of skin cancer, especially squamous cell carcinoma, and early ageing symptoms (photoageing) are long-term hazards.^30,31^ NB-UVB has been linked to xerosis, blistering, erythema and reactivation of herpes simplex.^32^

In clinical practice, PUVA may be preferred when durable remission is the primary therapeutic goal, particularly in patients with stage IA–IB MF. However, NB-UVB remains an appropriate option for patients requiring a safer profile, such as younger individuals or those with contraindications to psoralen. Future research should prioritize randomized controlled trials with standardized outcome definitions and long-term follow-up. Subtype-specific analyses, such as for folliculotropic MF, and evaluation of maintenance phototherapy protocols is also needed to guide optimized treatment strategies.

There are several limitations to this updated meta-analysis. Most included studies were retrospective with small sample sizes, heterogeneous patient groups and nonstandardized outcome definitions, resulting in moderate-to-low methodological quality. The high risk of bias and variability in study design limit the overall strength of our conclusions. Variability in patient adherence to treatment and baseline variations in attributes, including age, sex, comorbidities, and duration of illness before diagnosis and treatment were not taken into consideration in the pooled study.

Long-term outcomes and adverse effects were often under­reported, and maintenance phototherapy protocols, such as treatment frequency and duration, were not standardized across studies, limiting the consistency of results. In addition, results were not stratified by MF subtypes (e.g. folliculotropic MF), which may respond differently to therapy. Definitions of CR, PR and relapse varied, complicating direct comparisons. Moreover, variations in staging criteria, the absence of patient-reported outcomes, and limited geographical and ethnic diversity further constrain the generalizability of findings.

PUVA and NB-UVB are both effective treatment options for ­early-stage MF. PUVA was associated with a longer relapse-free interval, although complete and PR rates were comparable. Given the moderate-to-low quality and heterogeneity of the included studies, these findings should be interpreted with caution. PUVA may be considered when sustained remission is the primary therapeutic goal, but further high-quality randomized trials are needed.

## Supplementary Material

vzag055_Supplementary_Data

## Data Availability

All data are included in the article and its Supporting Information.
