# Supplementary material for: Comparison of narrowband ultraviolet B with psoralen plus ultraviolet A phototherapy for patients with early-stage mycosis fungoides: a systematic review and meta-analysis
Source: Skin Health Dis. 2026 Jun 15;6(4):406–14. doi: 10.1093/skinhd/vzag055 (PMC13425035; doi:10.1093/skinhd/vzag055)

**Supplementary**

**Figure 1.** PRISMA Flow Chart of Search Strategy for Present Systematic Review

**Figure 2.** Risk of Bias of Included Studies Assessed Using Cochrane Tools

**Figure 3**. Forest Plot of Partial Response in Early-Stage MF Treated by PUVA vs NBUVB

**Figure 4.** Forest Plot of Failed Response in Early-Stage MF Treated by PUVA vs NBUVB

**Figure 5.** Funnel Plot for Publication Bias of Meta-analysis of Any Response in Early-Stage MF Treated by PUVA vs NBUVB

**Figure 1.** PRISM flowchart of Search Strategy for Present Systematic Review

Studies included in previous version of review (n =7)

Reports of studies included in previous version of review (n =7)

Records identified from*:

(n =2,083)

PubMed (n =1,386)

Google Scholar (n =687)

Cochrane Library (n =10)

Records removed *before screening*:

Duplicate records removed

(n =693)

**Identification**

Total studies included in review

(n =9)

Reports of total included studies

(n =9)

Reports assessed for eligibility

(n =74)

Reports sought for retrieval

(n =80)

Records screened

(n =1390)

Records excluded**

(n =1310)

Reports not retrieved

(n =6)

**Screening**

Reports excluded:(n=65)

Non-comparative group

(n =45)

Comparison between PUVA vs INF-a (n =12)

Outcome of interest not reported (n=8)

New studies included in review

(n =2)

Reports of new included studies

(n =2)

**Included**

**Figure 2.** Risk of Bias of Included Studies Assessed Using Cochrane Tools


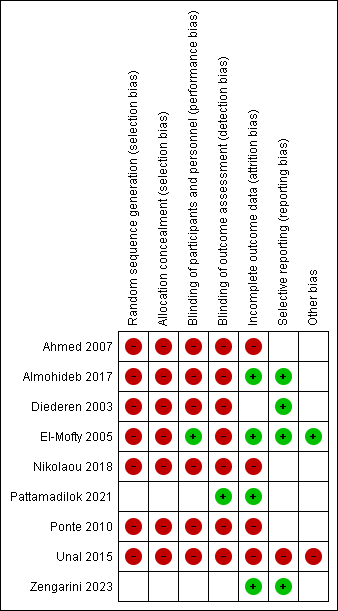


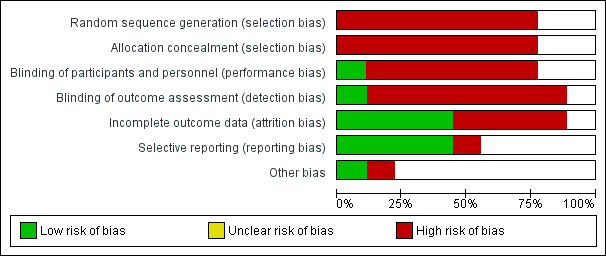


**Figure 3.** Forest Plot of Partial Response in Early-Stage MF Treated by PUVA vs NBUVB


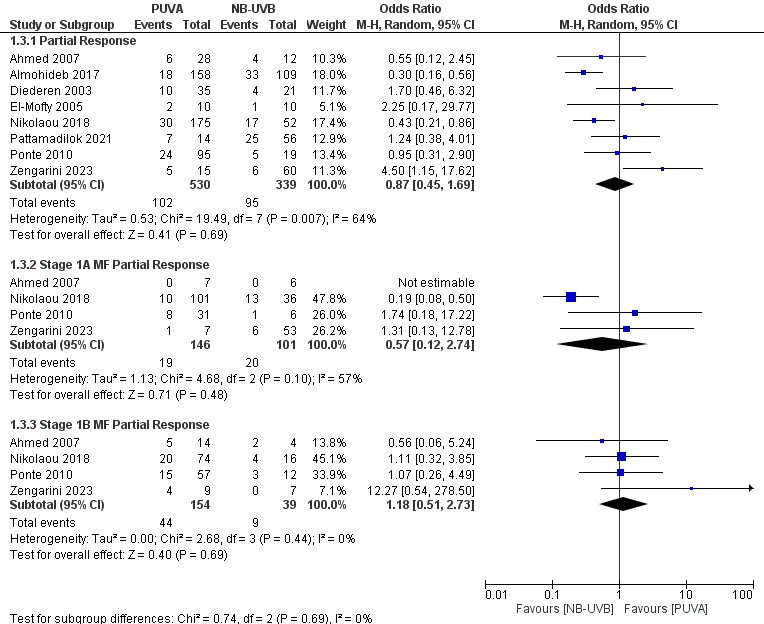


**Figure 4.** Forest Plot of Failed Response in Early-Stage MF Treated by PUVA vs NBUVB


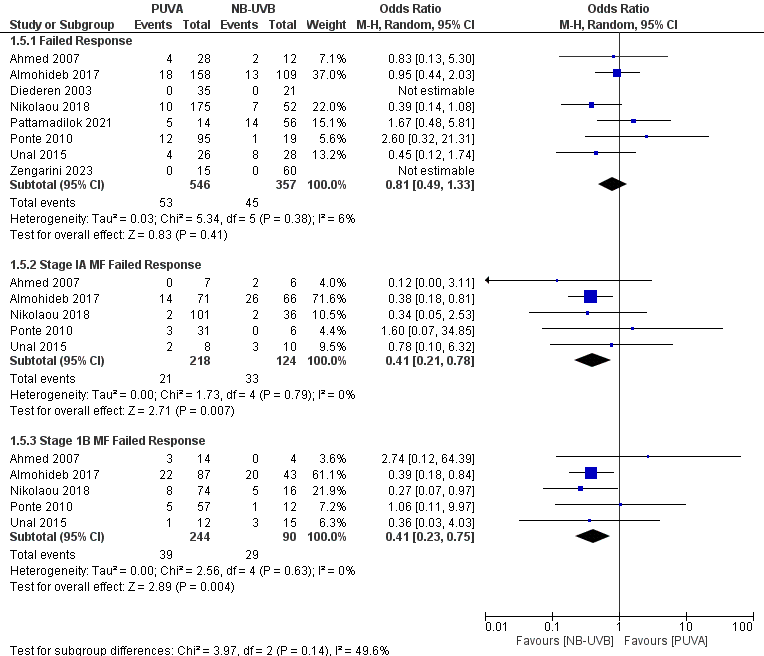


**Figure 5.** Funnel Plot for Publication Bias of Meta-analysis of Any Response in Early-Stage MF Treated byPUVA vs NBUVB


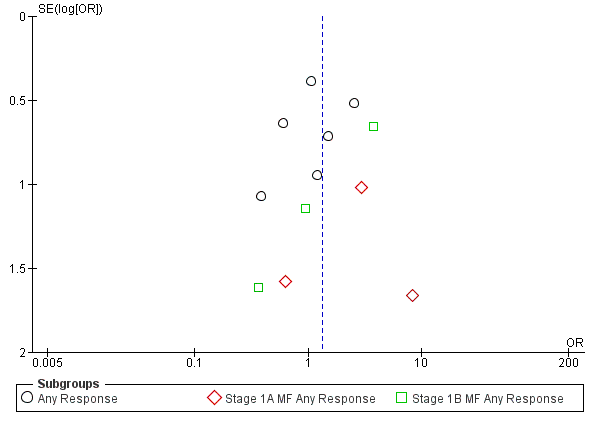

Supplement: vzag055_Supplementary_Data [file vzag055_supplementary_data.docx]
